# Nanoparticle Delivery in Prostate Tumors Implanted in Mice Facilitated by Either Local or Whole-Body Heating

**DOI:** 10.3390/fluids6080272

**Published:** 2021-08

**Authors:** Qimei Gu, Lance Dockery, Marie-Christine Daniel, Charles J. Bieberich, Ronghui Ma, Liang Zhu

**Affiliations:** 1 Department of Mechanical Engineering, University of Maryland Baltimore County, Baltimore, MD 21250, USA;; 2 Department of Chemistry and Biochemistry, University of Maryland Baltimore County, Baltimore, MD 21250, USA;; 3 Department of Biological Sciences, University of Maryland Baltimore County, Baltimore, MD 21250, USA;; 4 Marlene and Stewart Greenebaum Cancer Center, University of Maryland, Baltimore, MD 21201, USA

**Keywords:** gold nanoparticle, delivery to tumors, IFP, interstitial fluid pressure reduction, whole-body hyperthermia, local heating, ICP–MS, inductively coupled plasma mass spectrometry

## Abstract

This work discusses in vivo experiments that were performed to evaluate whether local or whole-body heating to 40 °C reduced interstitial fluid pressures (IFPs) and enhanced nanoparticle delivery to subcutaneous PC3 human prostate cancer xenograft tumors in mice. After heating, 0.2 mL of a previously developed nanofluid containing gold nanoparticles (10 mg Au/mL) was injected via the tail vein. The induced whole-body hyperthermia led to increases in tumor and mouse body blood perfusion rates of more than 50% and 25%, respectively, while the increases were much smaller in the local heating group. In the whole-body hyperthermia groups, the IFP reduction from the baseline at the tumor center immediately after heating was found to be statistically significant when compared to the control group. The 1 h of local heating group showed IFP reductions at the tumor center, while the IFPs increased in the periphery of the tumor. The intratumoral gold nanoparticle accumulation was quantified using inductively coupled plasma mass spectrometry (ICP-MS). Compared to the control group, 1 h or 4 h of experiencing whole-body hyperthermia resulted in an average increase of 51% or 67% in the gold deposition in tumors, respectively. In the 1 h of local heating group, the increase in the gold deposition was 34%. Our results suggest that 1 h of mild whole-body hyperthermia may be a cost-effective and readily implementable strategy for facilitating nanoparticle delivery to PC3 tumors in mice.

## 1. Introduction

Advancements in nanoparticle synthesis in the past two decades have broadened its potential medical applications and have had tremendous societal impacts. In recent years, utilizing nanoparticles as therapeutic carriers has revolutionized cancer therapy [1–4]. The incorporation of drug molecules into nanocarriers may protect a drug against degradation and offer the possibility of targeted and controlled release to specific tumors, thus considerably decreasing the incidence of deleterious side effects [5,6]. Nanoparticles have a large surface area-to-volume ratio, which allows for various entities to anchor onto the particle surfaces, including antibodies, drugs, and imaging agents [7]. Antibody attachments can be designed to specifically target receptors that are overexpressed on tumor cells [8]. While the aforementioned approaches have, in theory, led to prolonged circulation times and reduced systemic toxicity, only a very small fraction of the injected nanoparticles effectively enter tumor cells, resulting in a poor and heterogeneous drug delivery [9–12]. Among the possible transport barriers, a high interstitial fluid pressure (IFP) in a tumor is a major obstacle for drug delivery to tumors [13].

Transcapillary flow, which is crucial for the delivery of therapeutic agents, is regulated by the hydrostatic and colloidal osmotic pressures in capillaries and the interstitial space, together with the hydraulic conductivity and plasma protein reflection coefficient, as stated by Starling’s law [14]. Blood vessels within tumors lack fundamental structures and branching patterns when compared to those in healthy tissues [15]. The blood perfusion in tumors can be significantly weaker than that in normal surrounding tissue, and this arises from the compression and leakage of tumor blood vessels [13]. Unfortunately, a low hydrostatic pressure in tumoral capillaries is directly associated with a poor blood perfusion in the case of tumors. Furthermore, solid tumors show an increased IFP, which decreases the driving force of the transcapillary transport. Several factors may cause an increase in IFP within a tumor site, such as vessel abnormalities, fibrosis, shrinkage of the interstitial matrix, and a poorly formed lymphatic system. Vascular impairment may also be the result of cancer cell proliferation in a confined host tissue space [16]. Passive advection and the diffusion of nanoparticles are both negatively impacted by an elevated internal pressure and poor blood perfusion in tumors.

Blood flow is a major route of heat dissipation in hyperthermic conditions. Experimental studies have shown that blood flow in the skin increases by a factor of 4 upon heating at 43 °C for a period of 60 min [17]; however, heating-induced changes in the blood flow in some tumors have been found to be considerably different from those in normal tissue [17]. It has been shown that there was a limited increase in blood flow in tumors during the initial heating period. When the heating was prolonged or higher than 42 °C, the tumor blood flow progressively decreased [17]. The mild localized heating of tumors has been suggested to cause transient thermal damage to endothelial cells or alter cellular homeostatic mechanisms. This may lead to increases in transvascular permeability, thus boosting the local blood perfusion. A previous study by Hauck et al. speculated that a heating-induced increase in local blood perfusion might contribute to the observed enhancement of the delivery of chimeric ^125^I-labeled 81C6 in gliomas implanted in mice, after local heating at 41.8 °C for 4 h [18]. In the work of Lammers et al. [19], the effect of localized heating via immersing the tumor-bearing limbs of rats in a warm water bath for 1 h was evaluated. When compared to the control without heating, a statistically significant increase in copolymer delivery in one of the three studied tumor groups was reported. The authors speculated that hyperthermia increased the pore cutoff size of the vasculature to improve the accumulation of colloidal delivery in tumors; however, the effect was dependent on the type of tumor [19].

The experimental results demonstrating the effects of local heating on IFPs in tumors are not consistent. In one study, local heating was induced by immersing melanoma tumor-bearing Syrian golden hamsters in a water bath at 43 °C for a period of 30–60 min [20]. The authors reported significant decreases in tumoral IFPs and tumor growth rates [20]. A recent experimental study [21] reported reduced IFPs in human breast tumors, after the tumor temperature was elevated to 42 °C by laser heating, as well as an enhanced bulk accumulation of liposomes in the tumors [21]. However, no significant differences were seen at any time point when considering IFPs measured in implanted gliomas receiving local heating at 42 °C for several hours, compared with tumors in a control group [22].

Mild whole-body hyperthermia has also been used to enhance nanoparticle delivery to tumors. The effect of mild whole-body hyperthermia on the IFPs in tumors has recently been examined. For example, Sen et al. [23] and Winslow et al. [24] used mild whole-body hyperthermia with both murine colon tumors and murine melanomas implanted in mice. Their experimental studies demonstrated low IFPs in the tumors when the mice were subjected to several hours of whole-body hyperthermia, when compared to the controls without heating. Their experimental studies also established a correlation between IFP reductions, increases in local tumoral blood perfusion, and reductions in tumor hypoxia. Using similar approaches, our group performed experiments on PC3 human prostate cancer xenograft tumors implanted in immunocompromised mice [25]. We found significant reductions in the IFPs in tumors after 1 h of mild whole-body hyperthermia. MicroCT analyses of the resected tumors after an injection of a gold nanoparticle solution also suggested an enhanced nanoparticle delivery to the PC3 tumors in the heating group, which was 42% higher than that in the control group without heating [25].

In previous studies [17–25], the impacts of hyperthermia on drug delivery varied with the tumor type and depended on whether local or whole-body hyperthermia was applied. It is possible that temperature elevations in tumors and heating duration also played roles in the experimental observations. It was speculated that the observed consistent IFP reductions in previous whole-body hyperthermia studies [23–25] may be due to thermoregulatory responses, leading to changed flow patterns in the blood vessels upstream or downstream of the tumor vasculature, triggered by whole-body hyperthermia. One unanswered question is whether the local heating of tumors also induces IFP reductions in the same PC3 tumors exposed to temperature increases and a heating duration similar to those used during whole-body hyperthermia. In addition, it was not investigated in our previous study whether a prolonged whole-body heating of PC3 tumors would further reduce IFPs. Normal thermoregulation responding to elevations in body temperature suggests increases in blood perfusion to the superficial skin layer [17]. However, the extent of the enhancement of the blood perfusion rates in both the mouse body and implanted PC3 tumor was not measured in our previous experimental studies. Further investigation of the effect of the duration of whole-body or local hyperthermia is therefore warranted.

In this study, we investigated the effects of whole-body or local hyperthermia in PC3 tumors in lowering the tumoral IFP and increasing the blood perfusion rates of tumors and mouse body over a duration of 24 h post-heating. Through the tail vein of the mice, 0.2 mL of a previously developed nanofluid consisting of targeted gold nanoparticles was injected, with or without heating. During the experiments, the mouse body temperatures, tumor IFPs, and blood perfusion rates of the tumor and mouse body were measured at various time points. In addition, the nanoparticle accumulation in the tumors was quantified using ICP–MS (inductively coupled plasma mass spectrometry) on tumors resected 24 h post-heating.

## 2. Materials and Methods

### 2.1. Animals and Tumor Model

In one flank, 36 Balb/c Nu/Nu male mice (20–25 g) (Charles River, Frederick, MD, USA) were inoculated with 10^7^ PC3 human prostatic cancer cells. The PC3 cells were originally purchased from ATCC (Catalog#Crl-1435, American Type Culture Collection, Manassas, VA, USA). The tumor growth on the mice was monitored using digital calipers until the tumor reached a minimal transverse diameter of 10 mm (4–6 weeks), when the mouse weight varied between 28 and 32 g. The mice were randomly assigned to (1) the sham control group, without heating (*n* = 7); (2) the hyperthermia group, with 1 h of heating in a heating chamber (*n* = 15); (3) the hyperthermia group, with 4 h of heating in a heating chamber (*n* = 8); and (4) the hyperthermia group, with 1 h of local heating only on the tumor (*n* = 6). The animal experiment protocol (#LZ040971720) has been reviewed by the IACUC at the University of Maryland Baltimore County, and it was approved on 3 May 2017.

### 2.2. Experimental Measurements of Temperatures, IFPs, and Blood Perfusion Rates

**Figure 1.**
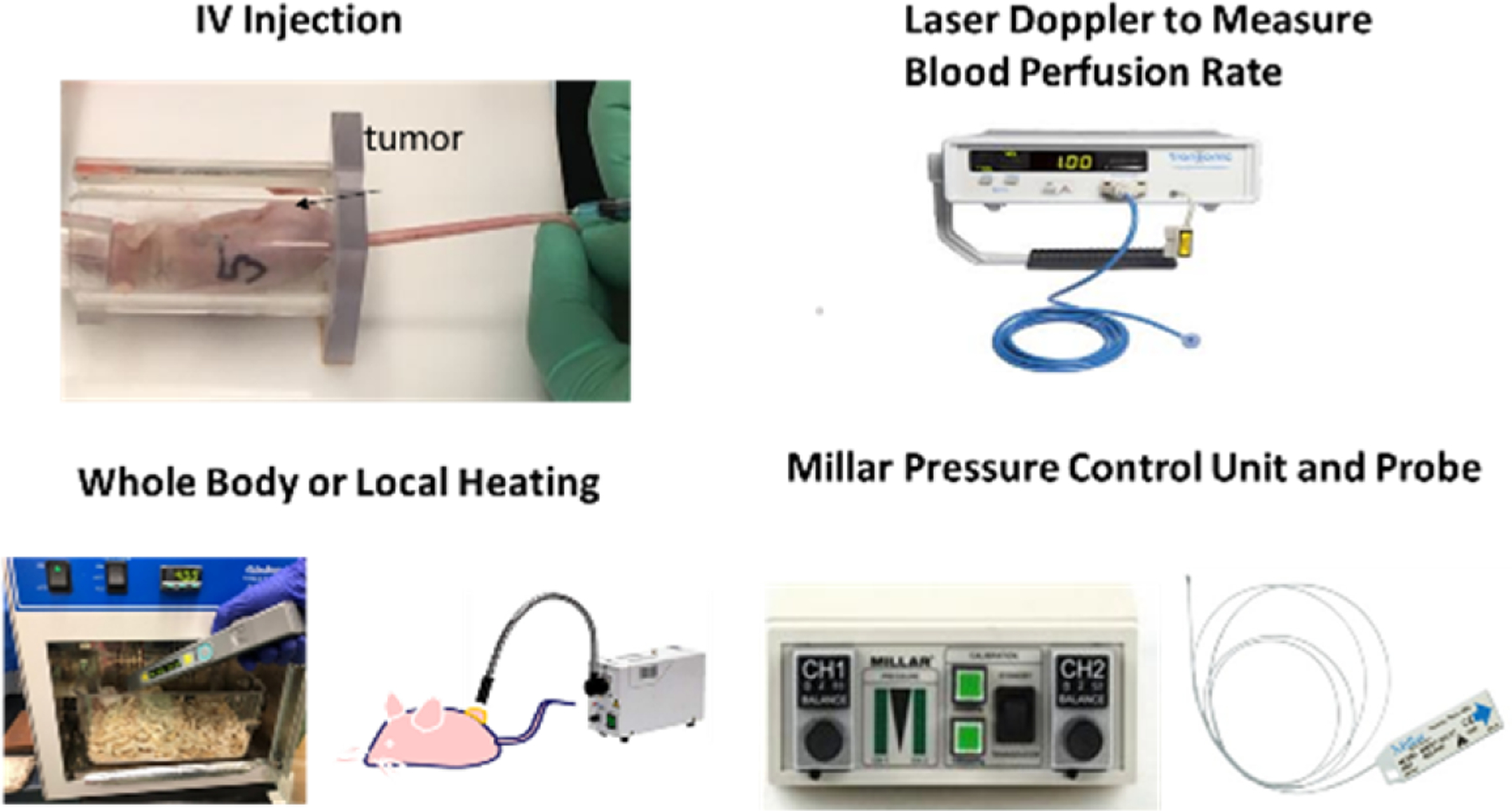
Equipment used in the experiments.

The mouse body temperature was monitored by a temperature reader (Bio Medic Data Systems, Seaford, DE, USA), implanted subcutaneously above the mouse shoulder 48 h before the experiment [25]. Mouse temperature measurements were conducted using a scanner to activate the temperature sensor (Figure 1). It should be noted that the temperature measured by the temperature reader is, in theory, 1 °C less than the actual mouse body temperature due to its superficial location.

During the experiments, the interstitial fluid pressure inside the tumor was measured using a micro-pressure transducer (Model SPR 524, Millar Instruments, Houston, TX, USA), with a catheter tip size of 0.33 mm (Figure 1). The IFP probe was calibrated using known hydraulic pressures in water columns. As in previous experiments [25], two needles were used: one for generating a track in the tissue, and the other for protecting the probe. The fragile pressure probe was inserted into one needle (26-gauge, Bevel tip, Fisher Scientific, Springfield, NJ, USA), with the probe tip advanced to the opening of the needle. Another identical needle was used before any IFP measurement to generate the same tissue track and allow for an easy insertion of the protection needle housing the IFP probe. After the track needle was withdrawn, the protection needle with the probe was inserted into the center of the tumor, and the pressure value was recorded after local equilibrium. Then, the protection needle with the probe was withdrawn towards the tumor periphery, and the IFP was measured at a tumor peripheral location halfway between the center and surface. For each tumor, the IFP was repeatedly recorded: baseline (before heating), immediately after heating, 2 h after heating, and 24 h after heating, along the same needle track. All the measurements were also performed on tumors in the control group, in parallel with the tumor in the experimental group, in order to evaluate whether insertion of the pressure probe in tumors alters the IFP readings.

The local blood perfusion rate was measured using a Laser Doppler Flowmeter (Tissue Perfusion Monitor BLF-22, Transonic System Inc., Ithaca, NY, USA). Figure 1 shows a flat Laser Doppler probe (BLF-22), which was attached to a skin surface to measure the local blood perfusion rate within a circular area of a diameter of 5 mm. A mouse skin surface location was selected to estimate the local blood perfusion rate of the mouse body, and the local blood perfusion rate of the tumor was measured by attaching the flat probe to the surface of the tumor. The blood perfusion rates were measured after pressure measurements. The Laser Doppler Flowmeter only gives a value with an arbitrary unit (AU); however, it can be used to evaluate local blood perfusion changes at the same skin location. It should be noted that the blood perfusion rates of the mice in the control group were also measured in parallel with the mice in the heating groups.

### 2.3. Experimental Protocols

**Figure 2.**
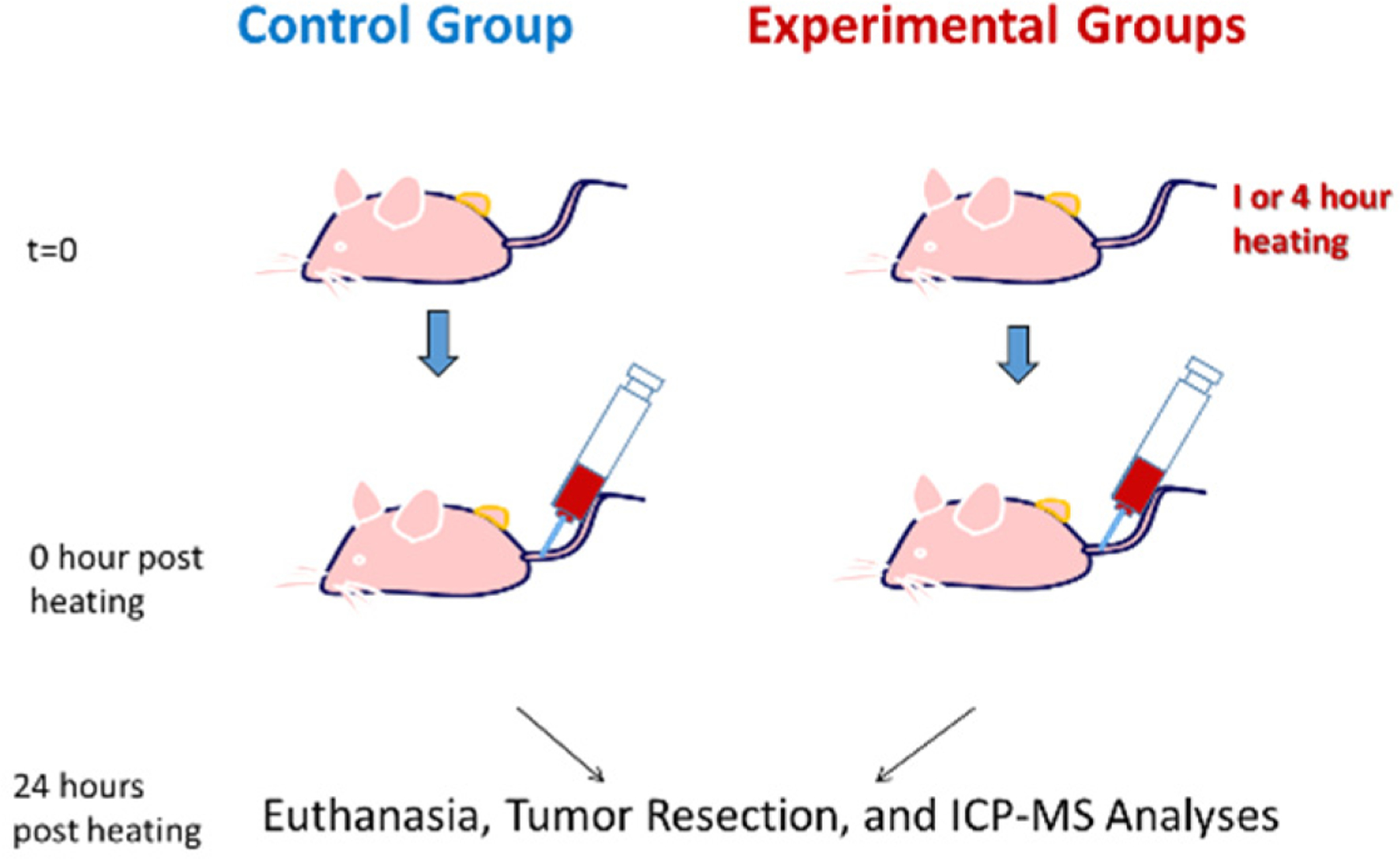
Schematic diagram of the experimental protocol.

The experimental protocols are shown in Figure 2. All mice in the whole-body hyperthermia groups were given an intraperitoneal (i.p.) injection of 1 mL of saline 30 min before heating to prevent dehydration. In the whole-body hyperthermia groups, the mouse was placed in a preheated cage within an incubator set at 39 °C. The mouse body temperature in the whole-body heating groups was measured every five minutes during the heating period. The cover of the incubator was open briefly for 5 s to allow for the insertion of the scanner to collect the temperature value. This only led to a slight decrease of approximately 0.1–0.3 °C in the air temperature inside the cage. In the local heating group, the tumor was heated with a fiber optic gooseneck microscope illuminator, which is shown in the schematic diagram in Figure 1. The mice in the local heating group were anesthetized using an isoflurane vaporizer, which was immobilized during the heating period. The mice were also placed on heating pads to maintain their normal body temperature. During the anesthesia, a 26-gauge needle containing a fine thermocouple (100 μm in diameter copper-constantan wires) was inserted into the center of the internal interface between the tumor and mouse body. The gooseneck illuminator was only used to heat the tumor. The intensity of the illuminator was adjustable, and preliminary studies were performed to determine its intensity to achieve a minimal temperature of 39 °C in the tumor, as measured by the thermocouple, while an infrared camera was used to confirm that the maximal temperature at the tumor skin was less than 41 °C.

After the heating, 0.2 mL of a previously developed nanofluid [22] was injected into the mouse circulatory system via the tail vein, as shown in Figure 1. The nanofluid was prepared using gold nanoparticles (AuNPs) functionalized with Fab fragments as active targeting moieties that specifically target the EphA2 receptor on PC3 cells (Fab fragment of the recombinant monoclonal antibody, 1C1). Specifically, the Fab fragment used was Fab-DBCO (Fab fragment derivatized with dibenzocyclooctyne for click-chemistry), provided by Dr. Ronald James Christie’s group at AstraZeneca (Gaithersburg, MD, USA) [26,27]. The details of the development of the nanofluid were described in our previous study [25]. Briefly, the nanoparticles were first coated with a polypropyleneimine (PPI) dendron displaying a thiol group at one end (for strong binding to the gold surface) and eight carboxylate groups at the other end (for covalent linkage to the linker connecting to the antibody fragment) [28]. This was achieved through a ligand exchange reaction by adding a large excess of the carboxylate-treminated dendron to a solution of citrate-coated gold nanoparticles under strong stirring at room temperature and overnight. An azide-terminated tetraethylene glycol spacer (H_2_N-TEG-N_3_) was then added to the dendritic coating of the nanoparticles using an EDC/sNHS coupling reaction. The DBCO-modified Fab fragment of 1C1 antibody was coupled to the dendronized gold nanoparticles using click chemistry in a PBS buffer. The finalized nanoparticles had an overall average diameter (gold nanoparticle core + Fab coating) of 28 nm in solution, as determined by dynamic light scattering (28 nm is the DLS size by number). The concentration of the injected nanofluid was 10 mg Au/mL. After the injection, the mice were later sent back to the animal facility. Twenty-four hours following the nanofluid injection, the mice were brought back to the lab and euthanized using a sodium pentobarbital overdose (160 mg/kg, i.p.).

After euthanasia, each tumor was resected, weighed, and immediately digested through a dissolution in acid for ICP–MS analyses [29,30]. Specifically, the tumor was dissolved in 10 mL of a 3:1 (*υ*/*υ*) solution of HNO_3_ (68%) and H_2_O_2_ (50%) and allowed to dissolve overnight. From this dissolved tissue solution, 0.5 mL was taken and mixed with 1.5 mL of freshly prepared aqua regia in a 50 mL conical tube. The tissue solution and aqua regia were well mixed and allowed to digest overnight. Then, the tissue/aqua regia solution (2.0 mL) was transferred to a glass volumetric flask and diluted to 100 mL. The resulting diluted solution was mixed thoroughly and used directly for ICMP–MS analysis (PerkinElmer^®^ NexION^®^ 300D ICP–MS system, Waltham, MA, USA). Calibration of the amount of gold in the tumor sample was conducted using a sample solution with a known gold mass that underwent the same preparation and analysis procedures.

### 2.4. Statistical Analyses

Descriptive analysis was conducted to provide measures of the central tendency and spread. The results in individual groups were calculated and are presented as high, low, median, and mean ± SD (standard deviation). Inferential analysis was performed via Student’s *t*-test to compare the results for the control and heating groups or the two heating groups. The one-way analysis of variance (ANOVA) was used to determine whether there was any statistically significant difference between the means of the two independent (unrelated) groups. A statistically significant difference between two groups was confirmed when the calculated *p*-value from Student’s *t*-test was less than 0.05.

## 3. Results

### 3.1. Tumor and Body Temperatures

**Figure 3.**
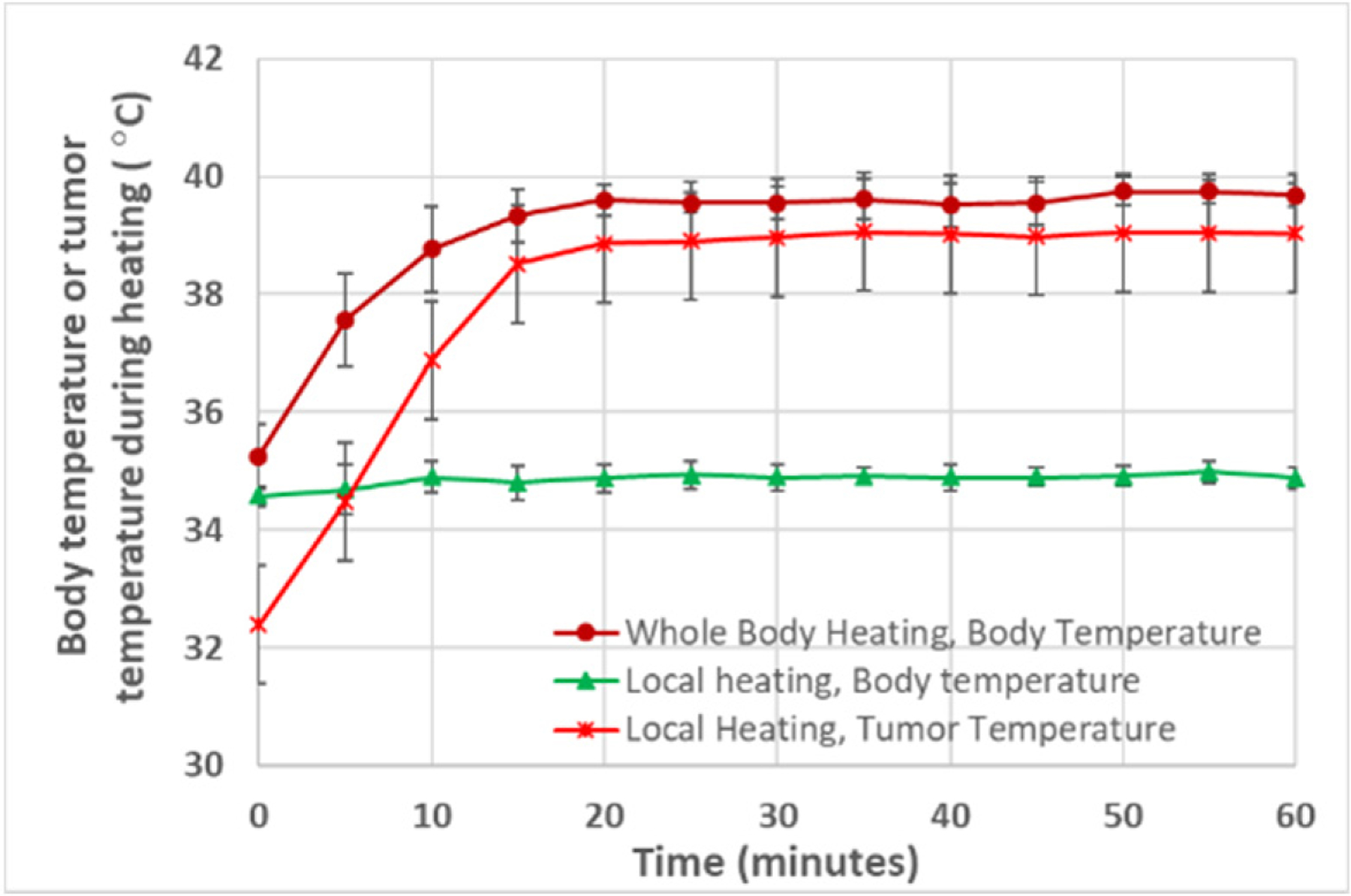
Typical temperature rising curves in the mouse body and tumor in the heating groups.

The mice behaved normally in the heating chamber and tolerated the 1 h or 4 h of heating well, with no observed adverse effects. However, the mice, after heating for 4 h, appeared more lethargic than the mice in the 1 h of heating group. In the group with local heating, using an illuminator to heat the tumor for one hour, we observed no adverse effects on the mice during and after the heating. As shown in Figure 3, in the whole-body hyperthermia groups, the body temperature increased quickly to 39.5 °C within 15 min and was maintained for the rest of the heating period. In the 1 h of local heating group, the body temperature of the mouse was maintained at its normal value, as shown in Figure 3. The minimal temperature within the tumor was monitored using a thermocouple, and it reached 39 °C after 15 min and remained at 39 °C for the rest of the heating period.

### 3.2. Blood Perfusion Rates in the Tumor and Mouse Body

**Figure 4.**
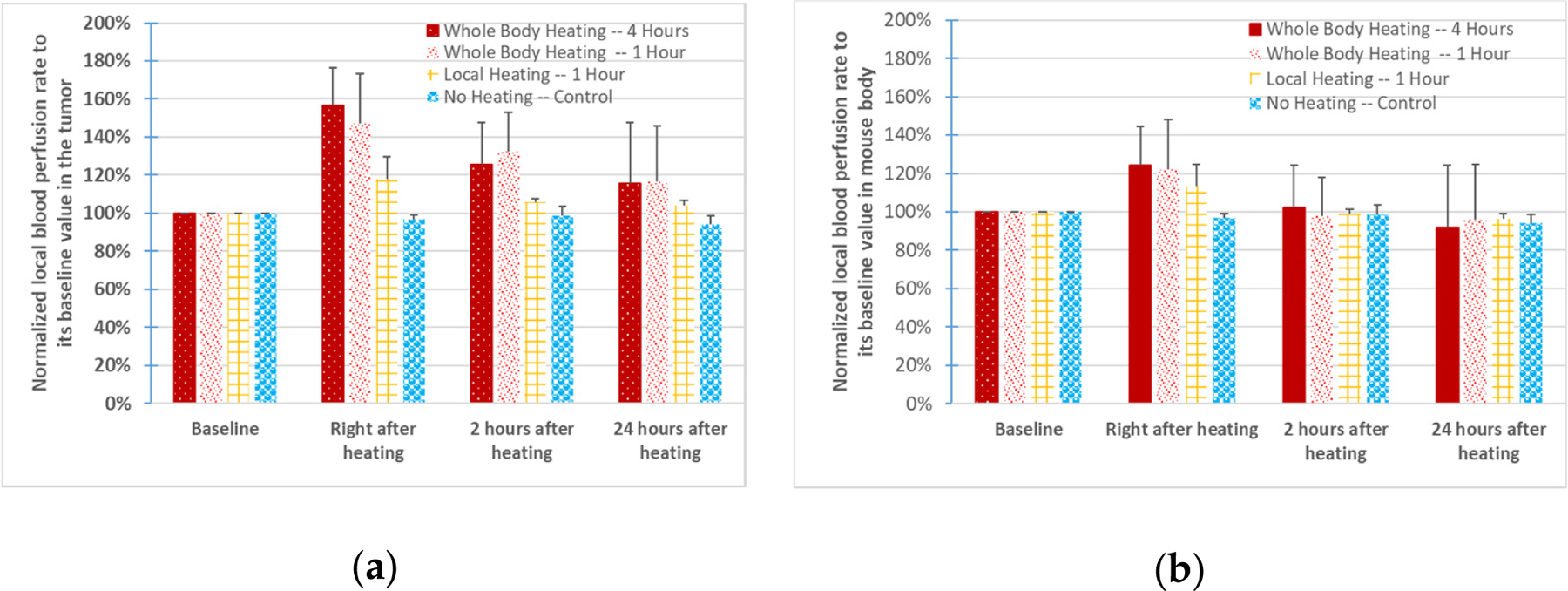
Local blood perfusion rates normalized to the baseline (before heating) values. Error bars represent the values of the standard deviation. (**a**) The left panel gives the values at the tumor site, and (**b**) the right panel shows the values for the mouse body.

The left panel of Figure 4 shows the mean and standard deviation values of the local blood perfusion rates, normalized to their baseline values. In the control group (first bar from the right), no significant changes in the blood perfusion rate were observed from one time instant to another. Immediately following the 1 h of whole-body heating (second bar from the left), the mean value and median value of the tumor blood perfusion rate were 150% and 149% of the baseline, respectively. At 2 h after the heating, they decreased slightly, but were still approximately 130% (mean) and 137% (median) of the baseline. At 24 h after the heating, the blood flow recovered almost to the baseline, remaining only 16% and 18% higher than the baselines for the mean value and median value, respectively. Similar increases in blood perfusion were found in the mice after heating for 4 h (first bar from the left). The 1 h of local heating (third bar from left) resulted in a small increase in the local blood perfusion rate, compared to whole-body hyperthermia, with only 18% and 21% for the mean value and median value, respectively. Two hours after local heating, the blood perfusion rate essentially recovered to its baseline level in the 1 h of local heating group.

Since the mean values of the blood perfusion increases are different among the groups, statistical analyses were performed to evaluate whether there were statistically significant differences caused by various factors. A *p*-value from comparing the results of the two groups using Student’s *t*-test was calculated to evaluate the confidence level of the significant differences. We first evaluated whether there was any statistically significant difference due to the different heating durations. As shown in the third column in Table 1, there was no statistical difference between the 4 h of whole-body hyperthermia group and the 1 h of whole-body hyperthermia group. However, *p*-values less than 0.05 were found when comparing the blood perfusion rate results from any whole-body hyperthermia group to the local hyperthermia group, right after heating (the fourth column in Table 1). We then evaluated whether the difference in the mean values of the blood perfusion rate between any heating group and the control group was statistically significant. The last column in Table 1 shows that the blood perfusion rate increases induced by whole-body heating were statistically significant when compared to the control group, immediately after heating (*p*-values: 0.004 and 0.0003, respectively) and 2 h post-heating (*p*-values: 0.045 and 0.007, respectively). When comparing the results of the local heating group to those of the control group, one finds that statistical significance was only found immediately after the heating, with a *p*-value equal to 0.0002, as shown in Table 1.

Whole-body hyperthermia increased the blood perfusion rate of the mouse body from the baseline value immediately after heating, with mean values of 21–25% and median values of 15–18%, as shown in the right panel of Figure 4. However, the increases were not sustained since they quickly recovered to the baseline value two hours after the heating. In contrast, the increase in the local blood perfusion rate of the mouse body in the local heating group was only 13% immediately after heating. As shown in Table 2, a statistical significance defined by a *p*-value less than 0.05 only occurred when comparing any of the heating groups to the control group right after the heating, with the *p*-value varying from 0.002 to 0.02.

### 3.3. IFPs in the Tumors

The baseline IFPs at the tumor center were scattered in all four groups (control: 5.1 ± 2.5 mmHg, whole-body heating for 1 h: 14.1 ± 6.8 mmHg, whole-body heating for 4 h: 9.9 ± 10.1 mmHg, and 1 h of local heating: 16.1 ± 3.1 mmHg). On average, the baseline IFPs at the tumor periphery were approximately 70% of that at the tumor centers. Since the baseline IFP varied significantly from one tumor to another, the IFP deviation from its baseline was used to evaluate whether local or whole-body hyperthermia decreased the tumoral IFPs.

In the control group (without heating), the IFP reductions at the tumor center varied from −4 to 3.2 mmHg, with a median of 0.5 mmHg. This implies that the insertion and withdrawal of the needle and pressure probe several times during the experiment did not result in significant changes in the IFPs in the control group. At the tumor center, the median values of the IFP reductions from their baseline in the 4 h and 1 h of whole-body hyperthermia group were 3 and 6 mmHg, respectively. However, the median values of the IFP reductions in the 1 h of local heating group were much smaller (approximately 1 mmHg from the baseline values).

**Figure 5.**
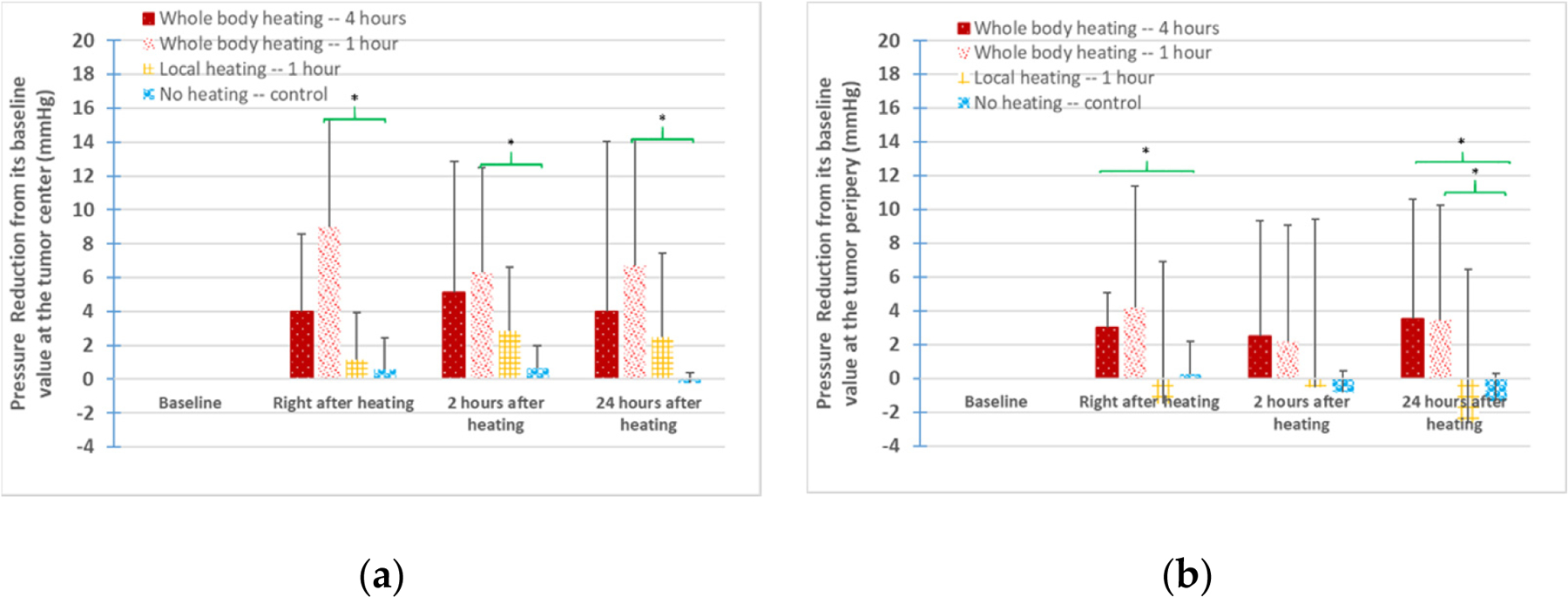
The mean values and standard deviations of the pressure reductions from the baseline values in the four tumor groups. (**a**) The left panel represents the IFP reduction at the tumor center, while (**b**) the right panel shows the IFP reduction at the tumor periphery. * denotes that a *p*-value < 0.05 between a heating group and the control group at the same time instant.

In Figure 5, the positive values indicate IFP reductions from the baselines, while the negative values indicate IFP increases. As shown in the left panel of Figure 5, the mean value of the IFP reductions from the baseline at the tumor center immediately after heating was 9.0 ± 6.3 or 4 ± 4.6 mmHg in the tumor groups with whole-body hyperthermia for 1 h or 4 h, respectively. Two hours post-heating, the IFP reductions increased in the 4 h of whole-body hyperthermia group, while they became smaller in the 1 h of whole-body hyperthermia group. The reductions in the IFPs also remained for over 24 h for all types of heating, as shown in the left panel of Figure 5. The IFP reductions at the tumor periphery location are illustrated in the right panel of Figure 5. The magnitudes of the IFP reductions were smaller than those at the tumor centers in the whole-body hyperthermia groups.

The IFP changes from the baseline values in the 1 h of local heating group were quite different from those in the whole-body hyperthermia groups (second bars from the right). Figure 5 shows that the mean values of the IFP reductions induced by local heating were less than 3 mmHg at the tumor center. However, at the tumor periphery location, the mean values of the IFPs actually increased, as shown by the negative values in the right panel of Figure 5. Therefore, considering both the tumor center and periphery locations together, one may conclude that the IFP reductions in the 1 h of local heating group were limited.

Statistical analyses were performed to evaluate whether the heating-induced IFP reductions were significant. As shown in Figure 5, although the averaged values of IFP reductions due to the 1 h of local heating were larger than those of the control group, the calculated *p*-values were all larger than 0.1. Large IFP reductions were observed in the whole-body hyperthermia groups. At the tumor center, only the IFP reductions in the 1 h of whole-body hyperthermia group were significantly different (*p* < 0.05) from those of the control group, as shown in the left panel in Figure 5. At the tumor periphery location (the right panel in Figure 5), *p*-values less than 0.05 were seen when comparing the values of the IFP reductions from either the 1 h or 4 h of whole-body hyperthermia group to those of the control group.

**Figure 6.**
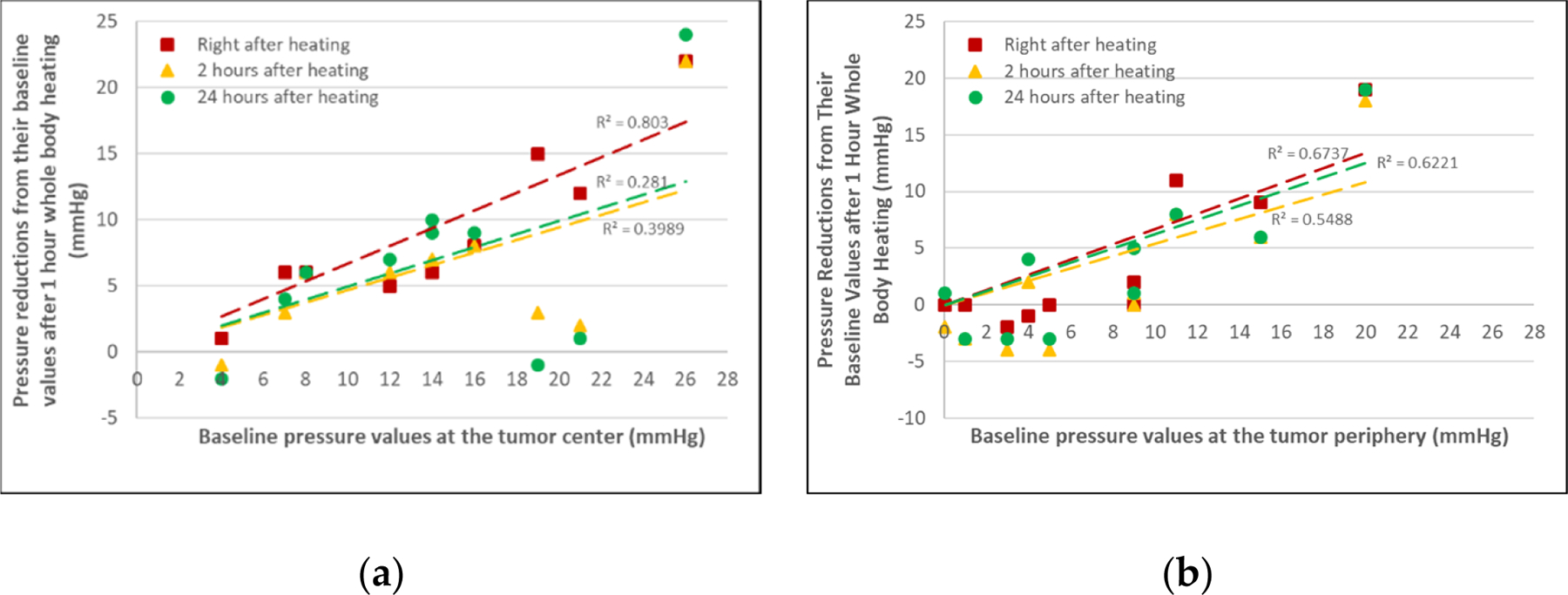
The relationship between the IFP reductions and their baseline IFPs in the 1 h of whole-body heating group. The data collected at various time instants post-heating are represented by different symbols. The lines are the linear curve fittings to the scatter data. (**a**) The left panel represents data at the tumor center, and (**b**) the right panel represents data at the tumor periphery.

Figure 6 shows the correlation between the IFP reductions and the baseline values. The baseline IFPs at the tumor periphery were smaller, varying from 0 to 20 mmHg (median: 9 mmHg), in comparison with those at the tumor center, with the IFP baseline values varying between 4 and 26 mmHg (median: 14 mmHg) in the 1 h of whole-body hyperthermia group. The left panel in Figure 6 shows the IFP reductions at the tumor center. The majority of the data (all but three) showed IFP reductions from the baselines, with a median value of the IFP reductions of 6 mmHg. Similar trends can be seen in the datasets for the tumor periphery location. As shown in the right panel in Figure 6, more than 35% of the measurements at the tumor periphery were negative, suggesting IFP increases from their baselines, although the majority of them (65%) were IFP reductions. The median value of the IFP reductions at the tumor periphery was 2 mmHg, which was smaller than that at the tumor center. While the data were scattered, the lines representing the curve fitting of the scattered data showed positive slopes. It may further be inferred that it is more likely to induce a larger IFP reduction in tumors with a higher baseline IFP.

### 3.4. ICP–MS Quantification

**Figure 7.**
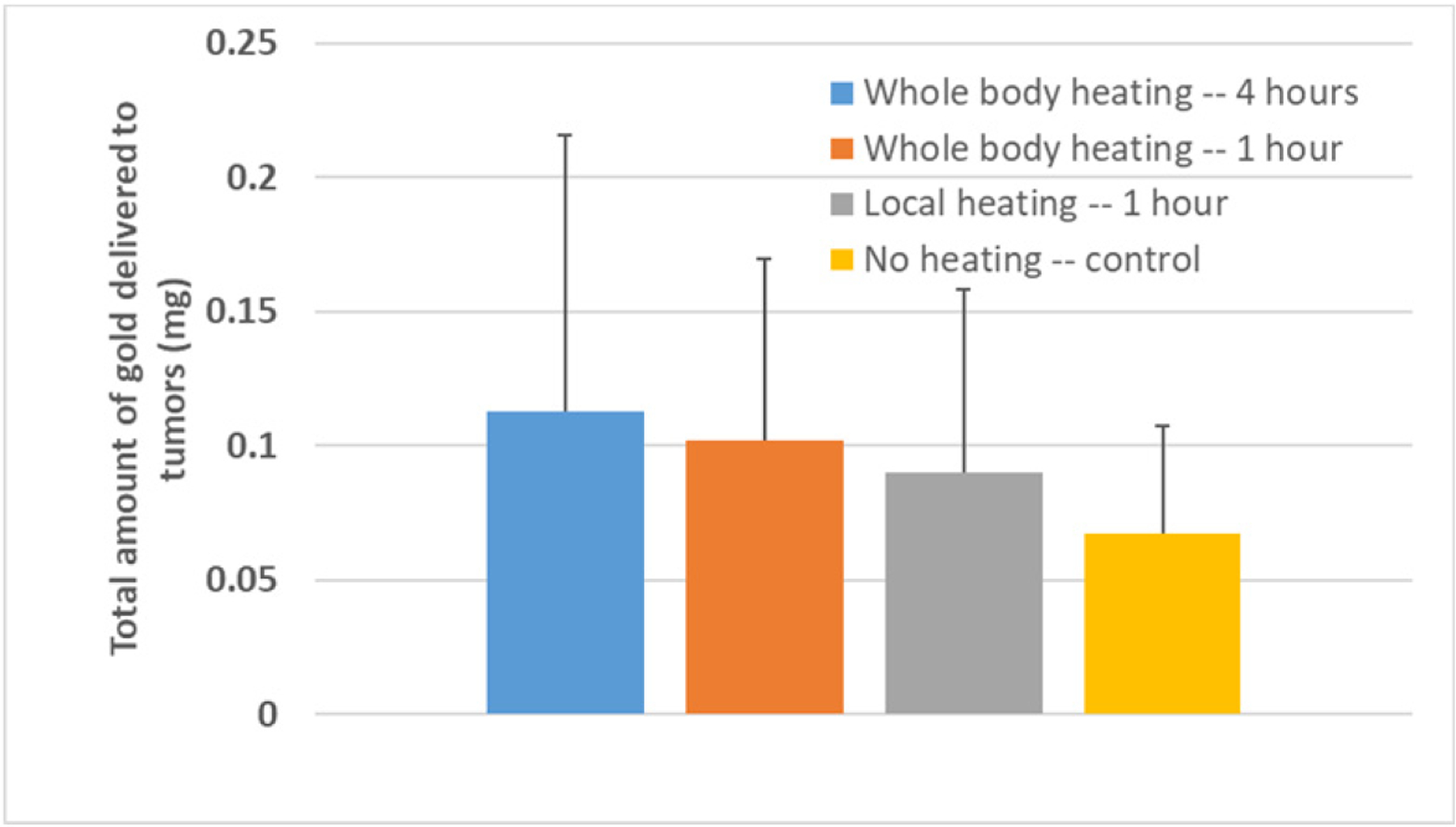
Mean values and standard deviations of the total amount of gold in the resected tumors in the control and heating groups.

The gold accumulation in the resected tumors was quantified using ICP–MS. The data showed large standard deviations within each group (Figure 7), which led to no statistical significance, when comparing any experimental group data with the data of the control group. The average amount of gold in the control group without heating was 0.0675 ± 0.0399 mg. Since the injected 0.2 mL of nanofluid (10 mg Au/mL) contains 2 mg of gold, on average, only 3.3% of the injected nanoparticles was deposited into the PC3 tumors. In the whole-body hyperthermia groups, on average, more than 5% of the injected gold nanoparticles accumulated in the tumors after the nanoparticles were circulating in the blood stream for 24 h. Compared to the control group, heating the mouse body for 1 h or 4 h resulted in an increase of 51% or 67% in the gold deposited in the tumors, respectively. In the 1 h of local hyperthermia group, the increase in the gold amount from the control group was only 34% on average.

## 4. Discussion

As suggested by previous studies [23–25], thermoregulation induced by the elevation of body temperature might contribute to IFP reductions and enhancements in therapeutic agent delivery to tumors. Thermoregulation is controlled by local and central mechanisms to return the body temperature to homeostasis [17]. Nerve cells scattered throughout the body in both the peripheral and central nervous systems are sensitive to changes in temperature. The collected temperature information is sent to the hypothalamus, thus maintaining a constant core temperature. Specific processes in thermoregulation might involve vasoconstriction in internal organs and vasodilation in the skin surface to deliver warm blood there and dissipate heat through the skin surface [17]. In the current study, the mice in the whole-body hyperthermia groups were awake, with their body temperatures elevated to 39.5 °C. We found increases of 50% in the blood perfusion rate in the PC3 tumors from their pre-heating levels, suggesting a possible activation of thermoregulation. The increase of 20% in the blood perfusion in the mouse body in the meantime implies a significant rise in the heart rate to maintain a normal systemic pressure, if one assumes that the stroke volume did not change. The heating time required to establish a steady state in the whole-body hyperthermia groups was approximately 15 min [25], and one would expect that it would take a similar time to cool the body down once the heating ends. A conservative estimate using heat transfer analyses suggests that the mouse body temperature might have returned to its normal value 2 h after the whole-body hyperthermia was ended. As shown in the current experiment, the blood perfusion rate of the mouse body recovered to its normal level 2 h post-heating. On the contrary, the blood perfusion rate in the tumors continued to be above its normal level for over 24 h. This may suggest abnormal properties of the tumor vasculature in reversible vasomotor control. This sustained a window of time to access the tumor vasculature and interstitial fluid space may be exploited to enhance the delivery of therapeutic agents, without concurrently increasing access to normal organs.

In the group with the 1 h of local heating applied to the tumor, the mice had to be immobilized to implement the surface heating, as well as to allow for the insertion of a temperature sensor. The mice were under anesthesia during the 1 h of local heating period. It is well known that general anesthesia suppresses thermoregulation [23–25]. In this study, a heating pad was used to maintain the normal body temperature of the mice. It seems that the 1 h of local heating only induced an increase of less than 18% in the blood perfusion rate of the tumor and a small increase in the blood perfusion rate of the mouse body (up to 13%). Two hours post-heating, both of the blood perfusion rates recovered to their normal levels. These measurements may suggest that the local heating alone was not sufficiently strong to trigger the sustained vasculature change observed in the whole-body hyperthermia groups.

The delivery of therapeutic agents to tumors depends on several major parameters. The transcapillary flow through the pores in the capillary walls is driven by the hydrostatic pressure in the tumor capillaries and the tumoral IFP [31]. Increases in the blood perfusion rate would result in an elevation in the hydrostatic pressure in the tumor capillaries. One can model the flow from capillaries to the venules as a simple flow resistance network. If one assumes that the hydrostatic pressure in the venules is close to zero, and the flow resistance of the blood vessel network is unchanged, one can easily determine that the hydrostatic pressure in the tumor capillaries would increase by 50% if the local blood perfusion rate were elevated by 50% [32]. This may be significant in amplifying the driven force to the transcapillary flow. The normal hydrostatic pressure in tumor capillaries is approximately 16 mmHg on average, which may be slightly higher or even lower than the tumoral IFP [31]. If this pressure increases by 50% to 24 mmHg, the resultant pressure difference across tumor capillaries would be dramatically larger, especially at the tumor center, when the IFP is elevated [31,32]. The large increase in the tumor blood perfusion rate in the three heating groups demonstrates positive impacts in the enhancement of the transcapillary flow rate, thus facilitating the delivery of nanoparticles to the interstitial space of PC3 tumors. Further, the size of nanoparticles is a factor that affects delivery to tumors. One challenge is the increased hydrodynamic diameter of nanocarriers loaded with therapeutic drugs. In this study, only one nanoparticle size was investigated. Future experiments should be conducted to evaluate how particle size affects transcapillary extravasation and diffusion into the interstitial fluid space.

In this study, we measured the IFP at each tumor location multiple times (before heating and several times after heating), unlike in previous studies [23,24], where the IFP was measured only once at each tumor location. Repeated measurements of IFP along the same path of the tumor, before and after heating, allowed us to minimize the uncertainty of IFP recordings at random tumor locations. When compared with the baseline values, the measured IFP reductions from the baseline values are evident in the whole-body hyperthermia groups. While the data were scattered, the majority of the measurements showed IFP reductions in the whole-body hyperthermia groups. When compared with the IFP changes in the control group, the *p*-values less than 0.05 suggested that the IFP reductions induced by whole-body hyperthermia were statistically significant. However, the IFP reductions in the 1 h of local heating group were much smaller than those in the whole-body hyperthermia groups. While the baseline IFPs were similar, the observed IFP reductions at the tumor center in the 1 h of local heating group were much smaller than those in the 1 h of whole-body hyperthermia group. At the tumor periphery, in contrast, the IFPs showed increases from their baseline in the 1 h of local heating group; therefore, on average, the IFP reductions in this group were very limited. Considering the lack of thermoregulation in the 1 h of local heating group, our results may suggest the important role played by thermoregulation in the large IFP reductions observed in the whole-body heating groups.

The specific mechanisms contributing to the IFP reduction are unclear. We speculate that thermoregulation triggered by systemic hyperthermia is a factor. As suggested by previous studies [23,24], during whole-body thermoregulation, systemically released neurovascular agents or signals may travel to the tumor to dilate various blood vessels, as an overall mechanism to dissipate heat to the surface of the body. Therefore, this may be directly related to the substantial and sustained increase in the blood perfusion rate to the tumor and the increased heart rate. IFP reduction may also be explained by an improved lymphatic drainage. In physical therapy, clinicians often suggest that patients take a hot shower to remove stagnant lymphatic fluid, thus increasing blood circulation [33]. A high solid stress in tumors has been observed in previous studies [31], which is attributed to the vessel compression and hypo-perfusion in tumors. If hyperthermia modifies the solid stress, some originally collapsed intra-tumoral lymphatic vessels may be reopened to improve fluid drainage [34,35]. A reduced solid stress may also contribute to decreases in the flow resistance across the tumor interstitial fluid space.

A theoretical simulation of fluid and nanoparticle transport in tumors has been performed since the 1980s. The tumor was typically modeled as a porous medium, and the transvascular flow was induced by distributed fluid sources and sinks of capillaries and lymphatic vessels. In the meantime, the nanoparticle concentration distribution in the porous tumor was determined by solving the continuum convection/diffusion equation, rather than modeling the motions of discrete nanoparticles. These theoretical simulations have been useful in evaluating proposed mechanisms to explain experimental observations. In Baxter and Jain [36], the effect of increases in the hydraulic conductivity of the microves-sels of a tumor on the drug/particle concentration distribution was investigated in a 1-D model of a spherical tumor. Additional theoretical simulations were conducted by El-Kareh and Secomb [37] to assess its dependence on the tumor size and shape. In recent years, these models were significantly improved by including the role played by cell uptake and particle elimination from the interstitial space, as well as incorporating a multiscale modeling approach [38] to evaluate the collective effect of nanoparticle attachments to tumor cells. Using these developed theoretical models, Stepleton et al. [39] showed the results of liposome transport in tumors similar to those in computed tomography (CT) experiments. The simulation results also illustrated that a decreasing steady-state IFP by only 1% was sufficient to increase the nano-therapeutic exposure [21]. As shown in recent theoretical simulations by our group [32], increases in the lymphatic drainage or the permeability of the porous tumor would lead to a significant decrease in the IFPs at the tumor center. The IFP reductions, combined with the local blood perfusion increases in tumors would result in the elevation of the nanoparticle concentration in the entire tumor region. A good agreement was found between the theoretically predicted and experimentally observed enhancement in nanoparticle delivery in tumors [32]. These theoretical studies suggested that increases in the hydraulic conductivity or recovery of lymphatic functions are possible mechanisms that lead to IFP reductions. More experimental studies are needed to continue to evaluate the roles played by individual mechanisms, especially with tools allowing for the real-time monitoring of those parameters.

Similar to previous studies, heating-induced blood perfusion increases and IFP reductions led to an enhanced delivery of therapeutic agents to tumors [23–25]. In our previous study, microCT was used to quantify the gold nanoparticle distribution and the total amount of gold deposited in resected tumors [25]. The microCT analyses in this study suggested an increase of 42% in the total amount of nanoparticles in PC3 tumors after 1 h of mild whole-body hyperthermia. The three-dimensional microCT image analyses also illustrated more nanoparticles delivered to the tumor center in the heating group than in the control group without heating. In the current study, ICP–MS was used to quantify the total amount of gold in resected PC3 tumors to evaluate the extent to which local or whole-body heating enhances the nanoparticle deposition in tumors. While the ICP–MS data were scattered in the current study, heating the mouse body for 1 h or 4 h resulted in an increase of 51% or 67% in the mean values of the amount of gold deposited in the tumors, respectively. The results are similar to those in our previous study using microCT analyses, and again, the results demonstrated possible correlations between an enhanced nanoparticle delivery to PC3 tumors and IFP reductions or increases in blood perfusion rates. In the 1 h of local heating group, the significant increase in the local blood perfusion rate alone may also be the primary factor that caused an increase of more than 30% in the nanoparticles deposited in the PC3 tumors.

There were some uncertainties associated with the accuracy of the ICP–MS quantified gold amount in the tumors in the current study. The uncertainties may be due to operational or instrumental errors during the preparation and quantification of the samples. ICP–MS is a quantification technique for measuring nanoparticle suspensions at very low concentrations [40,41]. Specimens are prepared by digesting tissue in mineral acids and diluting the resulting solution. The technique uses high-temperature plasma to vaporize and ionize the specimen, and the ions, such as gold ions, are directed to a mass spectrometer [42]. This method, if properly conducted, is a reliable technique in analytical chemistry. Previous reviews of ICP–MS suggest factors attributed to inaccuracy, including the heterogeneity of the particle concentration in the digested solution, storage time of the digested sample, and transport efficiency, which measures the conversion percentage of atoms into ions [40–43]. In the current study, although the resected tumors were digested in acid immediately after resection, the storage time of the solution varied due to restrictions in scheduling the time to access the ICP–MS system. Previous studies have observed that the measured gold mass fractions decreased with the increasing storage time. It was suggested that the loss of gold ions was caused by adsorption to the surfaces of the containers [43]. As shown in previous studies, the transport efficiency is the most influencing factor. The incomplete dissociation and ionization in plasma may be attributed to particle agglomeration/aggregation [40]. Not only does the transport efficiency deviate significantly from 100%, but its values also vary over time from one sample to another [29]. In the current study, we used a sample solution that contained the same gold nanoparticles with a known mass/concentration to establish a correlation between the measured intensity in ICP–MS and elemental mass quantity in the tested specimen solution. This is based on an assumption that the transport efficiencies of all the specimens of a single scan are the same as those in the calibration solution. This may minimize the variations in the transport efficiency from one scan to another. However, errors may still occur due to the assumed linear correlation, using the calibration solution to determine the slope of the linear curve. In addition, the total amount of gold deposited may not be the best method to quantify the nanoparticle delivery to tumors and draw conclusions. Using the gold amount per tumor weight or per blood flow to tumors may be a more informative approach to evaluate the effects of heating on particle delivery. Due to the current limitation relating to the small sample sizes, the factors that may be attributed to the uncertainties of the ICP–MS results could not be evaluated at this time.

While some comparisons with the control group showed significant differences in IFP reductions from the baseline values, the IFP data were scattered in the current study. However, as shown in the collected data, the majority of the measurements demonstrated IFP reductions, rather than increases, due to whole-body hyperthermia. On average, whole-body hyperthermia led to IFP reductions in tumors, even if the magnitude of the reduction varied significantly from one tumor to another. A possible reason for the large variation may be due to our selection of a 10 mm transverse diameter of the tumors to start the experiments. The size of the tumors may not be a good indicator of specific growth stages associated with the porous structures, leading to a large variation in the baseline IFPs. Future experimental studies are warranted to continue to investigate whether increasing the sample size would achieve more statistically significant IFP reductions.

This study may be helpful for future clinical studies focused on enhancing drug delivery to tumors. Currently, less than 4% of gold nanotherapeutic agents injected through the vein are deposited in the targeted solid tumors [11]. This implies that there is still room for improvement in enhancing drug delivery and minimizing systemic toxicity. There are many approaches to prolonging the circulation time of therapeutic drugs in the bloodstream or to improving the uptakes to tumor cells via an active targeting moiety on nanocarrier surfaces. Local or whole-body heating provides an alternative/additional method to significantly increase the delivery of drug-carrying nanostructures to targeted tumors. Among the three heating approaches, 1 h of whole-body hyperthermia may be the most cost-effective and readily implementable strategy for facilitating nanoparticle delivery to PC3 tumors in mice. Whole-body hyperthermia is relatively easy to implement and control, as compared to local heating. Temperature elevations in the tumors in the local heating group were not uniform, with variations of more than 2 °C in the tumors of 10 mm in diameter using an illuminator. A much larger temperature elevation would be expected in deep-seated tumors during local heating [44,45]. In addition, local heating may also cause collateral thermal damage to surrounding healthy tissue when the tumor is located in a deep tissue region [46]. In contrast, whole-body hyperthermia elevates the temperature of the arterial blood circulating the entire body. It typically results in a uniform temperature elevation in tumors when they are deep-seated. Among the two whole-body hyperthermia groups, heating the mouse body for 4 h did not further reduce IFP nor increase the blood perfusion rate when compared to the 1 h of whole-body hyperthermia group. The mice in the 4 h of whole-body hyperthermia group exhibited lethargy after the heating, although they tolerated the long duration of the heating. These results suggest that it is unnecessary to extend whole-body hyperthermia to more than 1 h, possibly due to the limit of thermoregulation, which reaches its upper bound after this heating duration. Future experimental studies are needed to investigate the minimal heating time needed to trigger thermoregulation in the body, as well as to evaluate whether elevating body temperature higher than 40 °C would shorten the time needed to enhance nanoparticle delivery.

## 5. Conclusions

We performed in vivo experiments on mice to compare the effects of local heating and whole-body hyperthermia in enhancing targeted nanoparticle delivery to PC3 tumors. The results showed consistent reductions in the IFPs of PC3 tumors after 1 h or 4 h whole-body hyperthermia treatments and significant increases in the blood perfusion rates in the tumor and mouse body after heating. Using ICP–MS analyses to quantify the total amount of gold in the resected tumors, we found that heating the mice resulted in an increase of 34% to 67% in the average amount of gold deposited in the tumors when compared to that in the tumor group without heating. We conclude that whole-body hyperthermia increases the blood perfusion rates and decreases the IFPs in PC3 tumors, while the effects of local heating are only evident in increasing the local blood perfusion rate. The ICP–MS quantification of gold in PC3 tumors may suggest an enhanced gold nanoparticle delivery, which may be the result of the observed perfusion increases and/or IFP reductions. Among the three heating groups, 1 h of mild whole-body hyperthermia may be a cost effective and readily implementable strategy to facilitate nanoparticle delivery to PC3 tumors in mice.

## Figures and Tables

**Table 1.** Calculations of the *p*-value of the two specific groups using Student’s *t*-test for the normalized blood perfusion rates in the tumors. It should be noted that the comparison of the two groups was conducted at the same time instance, i.e., 0 h, 2 h, or 24 h post-heating.

	Time Instances	1 h Whole-Body	1 h Local	Control
	0 h	0.32	0.03	0.004
4 h whole body	2 h	0.33	0.08	0.045
	24 h	0.48	0.18	0.42
	0 h	/	0.02	0.0003
1 h whole body	2 h	/	0.014	0.007
	24 h	/	0.14	0.40
	0 h	/	/	0.0002
1 h local	2 h	/	/	0.22
	24 h	/	/	0.31

**Table 2.** Calculations of the *p*-value of the two specific groups using Student’s *t*-test for the normalized blood perfusion rates in the mouse body. It should be noted that the comparison of the two groups was conducted at the same time instance, i.e., 0 h, 2 h, or 24 h post-heating.

	Time Instances	1 h Whole-Body	1 h Local	Control
	0 h	0.42	0.16	0.002
4 h whole body	2 h	0.31	0.35	0.32
	24 h	0.39	0.34	0.42
	0 h	/	0.20	0.02
1 h whole body	2 h	/	0.48	0.42
	24 h	/	0.48	0.43
	0 h	/	/	0.01
1 h local	2 h	/	/	0.39
	24 h	/	/	0.19

## Data Availability

The data are available in this article.
